# The effect of physical activity interventions on cognition function in patients with diabetes: A systematic review and meta‐analysis

**DOI:** 10.1002/dmrr.3443

**Published:** 2021-02-22

**Authors:** Ruitong Wang, Wenxin Yan, Min Du, Liyuan Tao, Jue Liu

**Affiliations:** ^1^ School of Public Health Peking University Beijing China; ^2^ Research Center of Clinical Epidemiology Peking University Third Hospital Beijing China

**Keywords:** cognition, diabetes mellitus, meta‐analysis, physical activity

## Abstract

**Background:**

In recent years, studies have revealed that cognition may be impaired by glucose metabolism disorder. Meanwhile, physical activity has been demonstrated to maintain blood glucose. This meta‐analysis was conducted to assess the effect of physical activity on cognition in patients with diabetes and provide evidence for the treatment of cognition impairment among them.

**Methods:**

We searched studies published in five databases from 1 January 1984 to 29 August 2020. A random‐effect or fixed‐effect meta‐analysis was used to estimate the pooled effect of physical activity on the change of cognition throughout intervention duration and post‐intervention cognition scores by standardized mean difference (SMD) and its 95% confidence interval (CI). We used funnel plots to evaluate the publication bias, *I* ^2^ statistic to evaluate the heterogeneity and did subgroup analysis stratified by sample size and follow‐up time.

**Results:**

Five eligible studies involving 2581 patients with diabetes were included. The pooled effect of physical activity on cognition improvement in patients with diabetes was significant (SMD = 0.98, 95% CI: 0.34–1.62), while the effect on post‐intervention cognition scores was not significant (SMD = 0.35, 95% CI: −0.04–0.73). In the subgroup analysis, the pooled effect was significantly higher in studies of follow‐up time less than 1 year (SMD = 2.14, 95% CI: 1.63–2.64), while observing no significant effect in studies of follow‐up time over 1 year (SMD = 0.10, 95% CI: −0.11–0.32).

**Conclusions:**

Physical activity is beneficial to improving cognition in patients with diabetes. However, the long‐term effect needs to be explored in future studies.

## 1 INTRODUCTION

Diabetes mellitus is a common chronic disease that features raised level of blood glucose due to the deficiency in insulin secretion or the inability for receptor to respond to insulin signal, which results in multiple complications including cardiovascular diseases, nerve damage, kidney damage, eye disease and cognitive dysfunction.^1^ ^,^ ^2^ With the change in people's lifestyle, the global prevalence of diabetes increases year by year. Currently, there is an estimation of 463 million adults aged 20–79 years old who live with diabetes, which constitutes 9.3% of the world's population.^2^ It is expected to reach 578 million cases (10.2%) in 2030 and 700 million (10.9%) in 2045, bringing heavy burden to families and the society.^2^

Currently, various epidemiological studies and scientific research have discovered the correlation between diabetes and cognitive dysfunction, demonstrating that patients with diabetes have lower cognitive function (including attention, memory and information processing speed) than healthy subjects while having a higher risk of dementia. It indicates that diabetes serves as a risk factor of cognitive impairment, which damages patients' memory and language function, declines their quality of life and leads to severe behaviour disorder.^3^, ^4^, ^5^, ^6^, ^7^, ^8^, ^9^, ^10^, ^11^, ^12^, ^13^, ^14^

Previous studies have revealed the positive influence of physical activity on both diabetes and cognitive impairment, showing that physical activity intervention not only contributes to hypoglycaemic control,^15^, ^16^, ^17^ but also reduces the risk of dementia and improves cognition function.^18^, ^19^, ^20^, ^21^ Furthermore, among studies which focused on the effect of physical activity intervention in patients with diabetes, some of them reported that physical activity had benefits on improving cognitive function in patients with diabetes,^22^, ^23^, ^24^, ^25^ while others did not find the impact of physical activity.^26^ The effect of physical activity intervention in patients with diabetes remains controversial.

Several meta‐analysis studies^27^ ^,^ ^28^ had been conducted to examine the effect of physical activity on the cognition of patients with dementia or the risk of cognitive impairment among patients with diabetes. However, no meta‐analysis has been made to synthesize the effect of physical activity on cognition in patients with diabetes. Patients with diabetes and complicated with cognitive dysfunction bring a much heavier burden for the care from family members, cause significant economic impact in the society, and increase the difficulty of treatment and compliance. Hence, we performed a systematic review and meta‐analysis of randomized control trials and cohort studies to systematically investigate the effect of physical activity on cognition function in individuals with diabetes.

## 2 MATERIALS AND METHODS

### 2.1 Data sources and search strategy

We searched for eligible studies published from 1 January 1984 to 29 August 2020, from five databases including PubMed, Embase, Web of Science, Medline and Cochrane Library using the following search term with no limitation of language: (‘physical activity’, ‘exercise’, ‘sports’, ‘walk’, ‘activity’, ‘danc(e/ing)’, ‘train’, ‘yoga’, ‘Tai Chi’, or ‘strength’) AND (‘trial’ or ‘RCT’ or ‘cohort’) AND (‘diabetes’ or ‘glucose’) AND (‘cognition’, ‘cognitive’, or ‘MMSE’). Records were managed by EndNote X 8.0 software to exclude duplicates. This study was strictly performed according to the Preferred Reporting Items for Systematic Reviews and Meta‐Analyses (PRISMA) and the PRISMA checklist was also provided in Appendix 1.

### 2.2 Inclusion and exclusion criteria

Included articles met the following criteria: (1) randomized control trials or cohort studies; (2) indicators about cognition influenced by physical activity could be obtained; and (3) diabetes were defined according to standardized guidelines, including American Diabetes Association guidelines, World Health Organization or International Diabetes Federation. Exclusion criteria were as follows: (1) studies irrelevant to the subject of the meta‐analysis (with participants without diabetes or without physical activity intervention); (2) insufficient data to calculate post‐intervention standardized mean difference (SMD) scores; (3) duplicated or overlapped articles; (4) reviews, editorials, conference paper or animal experiments.

Studies were identified by two investigators (WRT and YWX) independently according to the criteria above. Discrepancies were solved by a third investigator (DM).

### 2.3 Quality assessment

The quality of included studies was assessed by the criteria developed by Hoy and his colleagues.^29^ We assigned the 10 items with a total score of 10, with a score of 1 representing ‘yes’ while 0 represents ‘no’. According to the criteria, we assessed that the studies included had a moderate (6–8 scores) risk of bias. Two investigators (WRT and YWX) assessed the quality of studies independently, with discrepancies solved by a third investigator (DM).

### 2.4 Data extraction

Two researchers (WRT and YWX) scanned independently titles and abstracts of studies according to the inclusion and exclusion criteria to identify eligible studies, and full text was read if necessary. In the five studies selected, the following data were extracted independently by two investigators (WRT and YWX): (1) basic information including the first author and publication year of each study; (2) characteristics including sample size, mean age, sex ratio, cognition measurement method (scale), type of intervention, frequency, duration and therapy for control group; (3) primary outcomes including the scores and mean difference between control and intervention group's cognition, the mean difference of the change in cognition throughout intervention duration and the corresponding standard deviation.

### 2.5 Data synthesis and statistical analysis

We used a meta‐analysis to summarize data from RCT or cohort research and pooled the study‐specific estimates using a random‐effects or fixed‐effects model to obtain an overall summary estimate of the effect of physical activity across studies. The primary outcome in this study was the change of cognition throughout intervention duration. The secondary outcome was post‐intervention cognitive scores. The intervention effect was measured by the SMD of the change of cognition throughout intervention duration or post‐intervention cognition scores between intervention and control groups. The results of the included studies were performed with fixed‐effect models or random‐effect models in cases of significant heterogeneity between estimates. *I* ^2^ statistics was used to assess the magnitude of heterogeneity, with 25%, 50% and 75% representing low, moderate and high degrees of heterogeneity, respectively.^30^ The chosen proper effect model was based on the analysis results: the fixed‐effects model was used if *I* ^2^ ≤ 50% and the random‐effects model was used if *I* ^2^ > 50%.

If substantial heterogeneity was detected, we did subgroup analysis when possible to investigate the possible sources of heterogeneity using the following grouping variables: sample sizes and follow‐up time. Subgroup comparisons used the Q test. We considered a subgroup difference *p*‐value less than 0.05 to be indicative of significant difference between subgroups. Sensitivity analysis was performed by a deleted study with the lowest quality score and by using a different model (fixed‐effect or random‐effect model). The effect of physical activity was quantified using the SMD values and the corresponding 95% CIs, and a value of *p* < 0.05 was deemed significant. We used forest plots to describe the pooled effect of physical activity on related outcomes, and used funnel plots and Egger’ publication bias test to assess publication bias. We analysed data using Stata version 16.0.

## 3 RESULTS

### 3.1 Study selection and study characteristics

**FIGURE 1:**
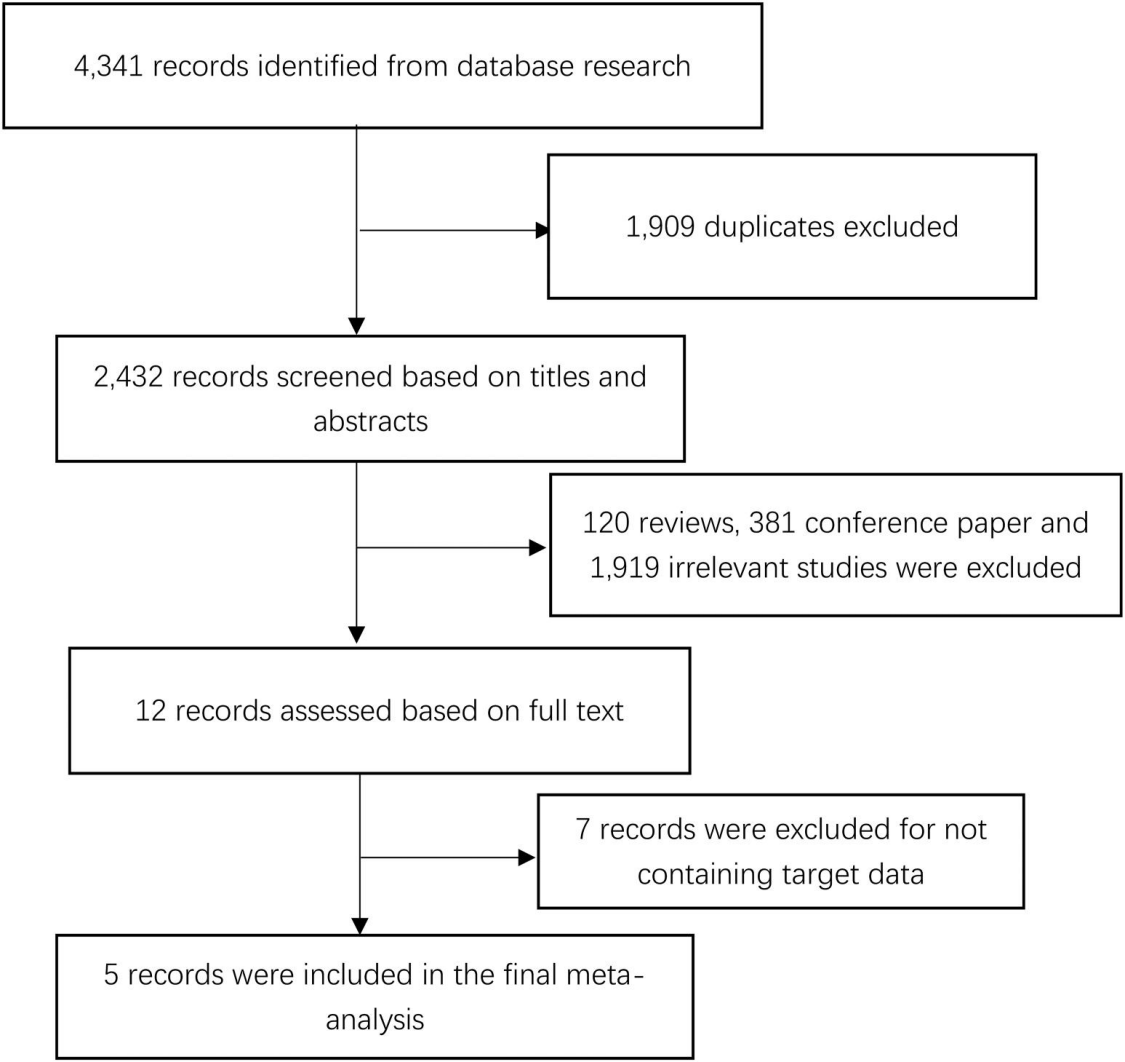
Flow diagram of study selection procedure

The systematic literature search identified 4341 articles. After screening the titles and abstracts of all references, 120 reviews, 381 conference paper and 1919 irrelevant studies were excluded, 12 potentially eligible articles were read full text, as 7 articles that failed to provide sufficient data or meet inclusion criteria were excluded. In total, five studies were therefore included (see Figure 1).

An overview of the included studies is shown in Table 1. The sample sizes varied from 47 to 1091 people with diabetes, resulting in a total of 2581 people with diabetes investigated, 1303 patients in intervention groups and 1278 patients serving as controls.^22^, ^23^, ^24^, ^25^, ^26^ Most studies were conducted in America (three studies), followed by one Australian study and one Chinese study. Four studies were RCT studies and one study was cohort study.

**TABLE 1:** Baseline characteristics of the five included studies

Study	Sample size	Age (mean)	Sex (male,%)	Cognition measurement method	Type of intervention	Frequency (min/week)	Duration	Therapy for control group	Type of study	Quality rating
Cai et al.^22^	50	63.96	43.64	MMSE	Aerobic exercise	90	12 weeks	Monthly telephone call	Cohort study	6
Callisaya et al.^25^	47	66.2	52.00	Cognitive global score^a^	Aerobic and resistance exercise	180	26 weeks	Stretching/gentle movement	RCT	6
Espeland et al.^24^	1091	58.32^b^	41.25	3 MSE	Aerobic exercise and diet modification	≥175	9.8 (8.4, 11.1) years	Education	RCT	6
Espeland et al.^23^	415	‐	37.35	3 MSE	Aerobic and non‐aerobic exercise	150‐200	2 years	Education	RCT	7
Espeland et al.^26^	978	‐	43.46	3 MSE	Aerobic exercise and diet modification	≥175	8.1 (7.8 ,9.3) years	Education	RCT	7

Abbreviation: RCT, randomized controlled trial.

^a^ The cognitive global score was formed as a composite of the following tests calculated as *z* scores standardized to the baseline mean and SD: (1) the Victoria Stroop test (interference score C‐D); (2) the Trail Making Test (shifting score B‐A); (3) the Digit Symbol Coding Test (DSC); The digit span subtest of the Wechsler Adult Intelligence Scale – Third Edition (WAIS‐III); Controlled Oral Word Association Test (COWAT); The three‐part Hopkins Verbal Learning Test – Revised (HVLT) and the Rey Complex Figure copy and delay.

^b^ After excluding the drop‐outs.

### 3.2 Effects of physical activity interventions on cognitive function in patients with diabetes

**FIGURE 2:**
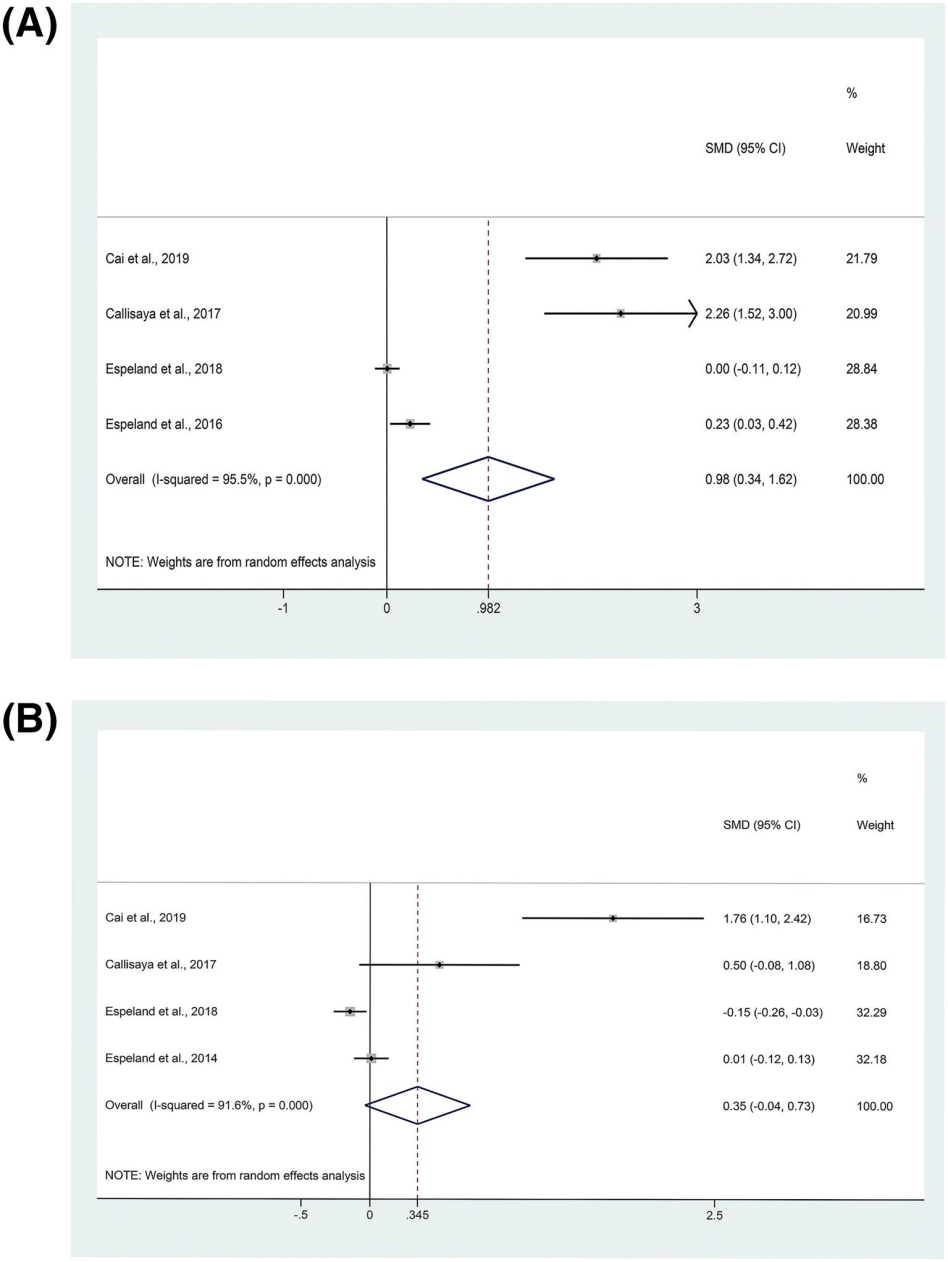
Forest plots for effect of physical activity on cognition of patients with diabetes: (A) The change of cognition throughout intervention duration. (B) Post‐intervention cognitive scores

The pooled effect of physical activity on the change of cognition throughout intervention duration and post‐intervention cognition scores are shown in Table 2. The pooled analysis showed a significant effect of physical activity interventions on the improvement of cognition function (SMD = 0.98, 95% CI: 0.34–1.62, *I* ^2^ = 95.5%, *p* = 0.003, four studies (Cai et al., 2019; Callisaya et al., 2017; Espeland et al., 2018; Espeland et al., 2016), 1 study (Espeland et al., 2014) excluded due to lack of data, Figure 2A). We observed no significant effect of physical activity interventions on the post‐intervention cognitive scores (SMD = 0.35, 95% CI: −0.04–0.73, *I* ^2^ = 91.6%, *p* = 0.076, four studies (Cai et al., 2019; Callisaya et al., 2017; Espeland et al., 2018; Espeland et al., 2014), one study (Espeland et al., 2016) excluded due to lack of data, Figure 2B).

**TABLE 2:** Relative outcomes of the five included studies

		Change of cognition throughout intervention duration			Post‐intervention cognitive scores (mean [SD])		
Study	Sample size(N) Intervention/Control	Intervention (mean [SD])	Control (mean [SD])	SMD (95% CI)	Intervention (mean [SD])	Control (mean [SD])	SMD (95% CI)
Cai et al.^22^	27/23	2 (1.29)	−0.18 (0.744)	2.03(1.34,2.72)	28.85 (1.05)	26.91 (1.16)	1.76 (1.10,2.42)
Callisaya et al.^25^	24/23	0.14 (0.066)	−0.01 (0.066)	2.26(1.52,3.00)	0.2 (0.539)	−0.08 (0.576)	0.50 (−0.08,1.08)
Espeland et al.^24^	554/537	−0.044 (0.471)	−0.046 (0.463)	0.00(‐0.11,0.12)	0.177 (0.682)	0.277 (0.695)	−0.15 (−0.26,‐0.03)
Espeland et al.^23^	199/216	0.028 (0.663)	−0.121 (0.661)	0.23(0.03,0.42)			
Espeland et al.^26^	499/479				92.25 (5.94)	92.19 (6.66)	0.01 (−0.12,0.13)

Abbreviations: CI, confidence interval; SD, standard deviation; SMD, standardized mean difference.

### 3.3 Sensitivity analysis and publication bias

**FIGURE 3:**
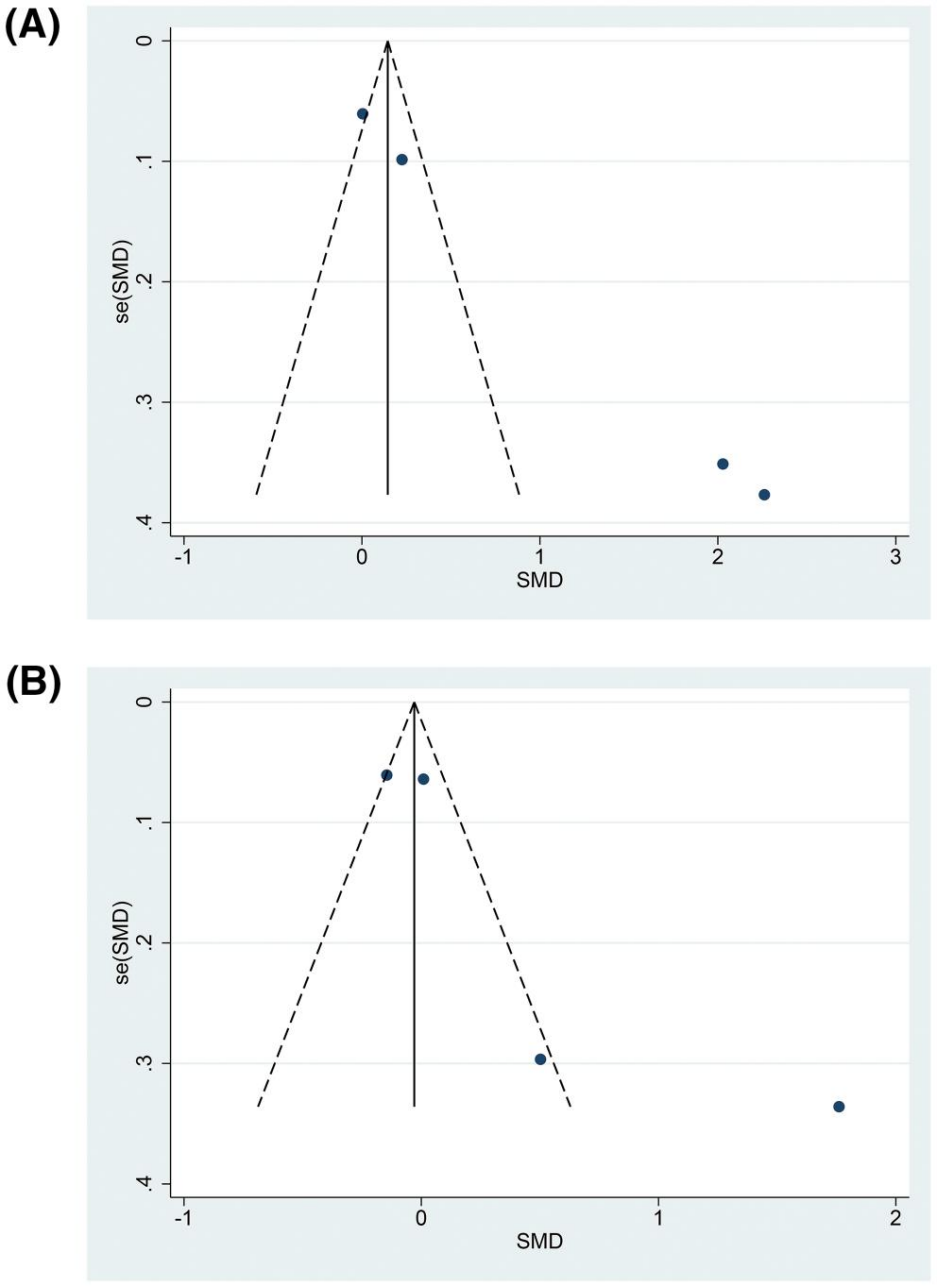
Funnel plots for effect of physical activity on cognition of patients with diabetes: (A) The change of cognition throughout intervention duration. (B) Post‐intervention cognitive scores

In the sensitivity analysis, the pooled results of meta‐analysis using random‐effect models were consistent with the principal findings of fixed‐effect models or deleting study of the lowest literature quality score, which showed that the results of pooled effect (SMD) of physical activity interventions on cognitive function were stable. Both funnel plot and Egger's test showed publication bias on the pooled effect of physical activity interventions on change of cognition (*p* = 0.001, Figure 3A), while there was no evidence of publication bias on the effect of physical activity interventions on the post‐intervention cognitive scores (*t* = 2.57, *p* = 0.124, Figure 3B).

### 3.4 Subgroup analysis

In the subgroup analysis, the pooled effect of physical activity on improvement of cognition function was significantly higher in studies of follow‐up time less than 1 year (SMD = 2.14, 95% CI: 1.63–2.64, *I* ^2^ = 0.0%, *p* = 0.651), while there was no significant effect on cognition in the study of follow‐up time more than 1 year (SMD = 0.10, 95% CI: −0.11–0.32, *I* ^2^ = 72.5%, *p* = 0.056).

## 4 DISCUSSION

This is the first meta‐analysis to assess the effect of physical activity on cognition function among patients with diabetes. In this systematic review and meta‐analysis, we included five studies related to physical activity, diabetes and cognition. Our results showed that the pooled effect of physical activity intervention on improving cognition function was significant (SMD = 0.982, 95% CI = 0.342–1.622) among patients with diabetes. Groot et al.^27^ made a meta‐analysis of 18 RCT studies and showed that physical activity interventions positively influenced cognitive function in all patients with dementia. Zhang et al.^28^ made a meta‐analysis of 17 studies and showed that the risk of Alzheimer's disease (AD) is higher among people with diabetes than in the general population, they suggested that the necessary treatment measures should be taken in order to decrease the risk of AD. This was similar with the results from our study that physical activity positively improved cognition of patients with diabetes. Our findings indicated that physical activity could be a measure to prevent AD and reduce the risk of dementia in individuals with diabetes.

Diabetes is an important public health problem. Uncontrolled diabetes could lead to complications in many organs including brain. Previous studies reported that the presence of comorbidities in severe acute respiratory syndrome patients increased the risk of death by nearly twofold.^31^ Balance and working memory functions were simultaneously impaired in patients with type 2 diabetes.^32^ In contrast, physical activity was associated with less microvascular disease in the brain and in other vascular beds^33^ and less brain atrophy.^34^ Previous studies reported that physical activity could improve cognition in patients with dementia and diabetes. It is noteworthy that individuals with diabetes, who experienced greater cognitive difficulties, were less likely to remain adherent to exercise or diet.^35^ So it was recommended for clinical that physical activity intervention should be undertaken as early as possible.

Among the included studies, physical activity intervention focused on aerobic and resistance training such as walking, strength, flexibility, balance training and qigong (a Chinese kung fu), 3–4 times per week and 30–50 min at a time. An RCT of 112 participants by Haritz Arrieta, et al.^36^ reported that a 6‐month individualized, progressive, multicomponent physical exercise intervention is effective at maintaining cognitive function. Miu et al.^37^ found no statistically significant difference between the two groups (aerobic exercises versus medical treatment) with respect to cognitive function by an RCT of 85 patients with dementia in Hong Kong.

Physical activity is one of the major components of the non‐pharmacological interventions in glycaemic control for patients with diabetes. The presently used drugs in AD therapy show a temporary benefit only in the early stage of the disease.^38^ There is no specific cure, so for patients with diabetes combined with cognitive impairment, it is particularly important to take physical activity and other non‐drug interventions in early stage to prevent the deterioration of AD. Esteban‐Cornejo et al.^39^ performed analyses with Cox regression on 3677 individuals from Spain aged 60 years or older. They found that the relationship between cognitive vulnerability and inactive elderly mortality was more significant, and activity reduced the mortality of cognitively vulnerable individuals by 36%. These novel results highlight that physical activity could improve the survival of older adults with cognitive impairment.

A possible explanation for the impact of physical activity on cognition is that AD is marked by changes in cerebral blood flow (CBF); patients with AD show a 40% decrease in global blood flow compared with healthy controls. Increasing physical fitness by aerobic exercise assists in the prevention or slowing of pathological cognitive decline by an increase in CBF.^40^ Another possible physiological mechanism is about β‐hydroxybutyrate (β‐HB), which is synthesized in the liver and transported to the body through blood circulation, is an intermediate product of fat metabolism. It is able to pass through the blood–brain barrier and becomes one of the major energy sources in the brain. β‐HB has neuroprotective effects, which alleviates neurodegenerative disease by improving mitochondrial functions. β‐HB also promotes the expression of brain‐derived neurotrophic factor (BDNF), an important neurotrophic factor that is associated with synapse plasticity and adult hippocampal neurogenesis. A study of Lan et al.^41^ showed that aerobic exercise increased the levels of β‐HB and the BDNF transcript; they suggested that aerobic exercise improved adult hippocampal neurogenesis and cognition function, probably through increasing β‐HB‐induced BDNF transcription. Myokines produced by skeletal muscle were systemic factors of blood flow that contribute to the beneficial effects of physical activity on the brain.^42^ In recent years, systemic factors released by skeletal muscle, such as FNDC5 and its irisin secretion, have attracted attention as beneficial exercise regulators for the brain. Interestingly, Young et al.^43^ identified FNDC5 as an important regulator of BDNF and endurance exercise induced irisin expression not only in skeletal muscle but also in the hippocampus, a brain region involved in memory and spatial awareness, indicating that physical activity can improve cognition through systemic factors associated with blood flow.

**FIGURE 4:**
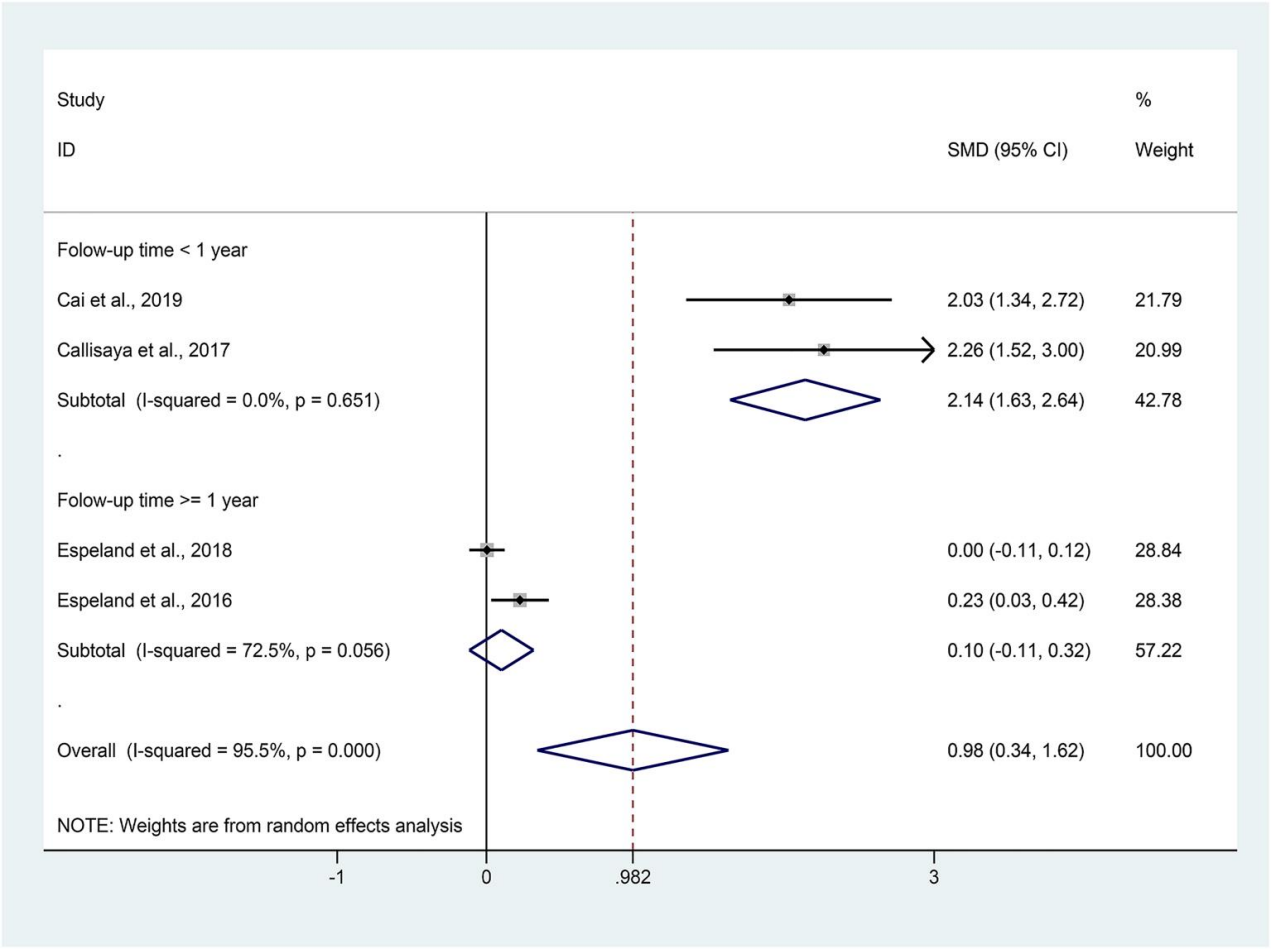
Forest plots for subgroup analysis of the effect of physical activity on the change of cognition function in patients with diabetes

The results of subgroup analysis revealed that the pooled ameliorative effect of physical activity interventions was higher in studies less than 1 year of follow‐up (2.14; 95% CI: 1.63–2.64, Figure 4), while there was no effect on another group (follow‐up time ≥1 year) (0.10; 95% CI: −0.11–0.32, Figure 4). We speculated that the adherence of participants was worse with longer follow‐up time and cognition function of individuals with diabetes tend to worsen with the time of degradation. So, it is of great significance to carry out physical activity intervention on patients with diabetes earlier.

There are several limitations in this study. First, publication bias exists in this meta‐analysis. Second, our findings may be somewhat limited by the difference of cognitive tests and variation in cognitive tests may have affected the accuracy of the effect of the intervention. Another potential limitation is the variability in patient populations across studies. More adequate and vigorous research should be conducted to prove the associations found in this study.

## 5 CONCLUSION

This meta‐analysis shows that physical activity interventions including aerobic exercises are beneficial to improving cognitive function in patients with diabetes. Our findings could provide evidence for physicians regarding the efficacy of physical activity in patients with diabetes to improve cognitive function and prevent dementia. However, the long‐term effect of physical activity intervention needs to be explored in future studies.

## CONFLICT OF INTEREST

The authors declare no potential conflicts of interest.

## AUTHOR CONTRIBUTIONS

Jue Liu conceived and designed the study; Wenxin Yan and Ruitong Wang carried out the literature searches; Wenxin Yan and Ruitong Wang extracted the data; Wenxin Yan, Ruitong Wang and Min Du assessed the study quality; Wenxin Yan and Ruitong Wang performed the statistical analysis; Ruitong Wang and Wenxin Yan wrote the manuscript; Jue Liu, Min Du and Liyuan Tao revised the manuscript.

### PEER REVIEW

The peer review history for this article is available at https://publons.com/publon/10.1002/dmrr.3443.

## Supporting information

Supplementary Material

## Data Availability

The data of this study are available from the corresponding author upon request.
